# Development of “Yima Nkqo,” a community-based, peer group intervention to support treatment initiation for young adults with HIV in South Africa

**DOI:** 10.1371/journal.pone.0280895

**Published:** 2023-06-15

**Authors:** Maria F. Nardell, Siyaxolisa Sindelo, Elzette Rousseau, Nomakaziwe Siko, Pamela Fuzile, Robin Julies, Ingrid V. Bassett, Claude A. Mellins, Linda-Gail Bekker, Lisa M. Butler, Ingrid T. Katz

**Affiliations:** 1 Division of Global Health Equity, Brigham and Women’s Hospital, Boston, MA, United States of America; 2 Harvard Medical School, Boston, MA, United States of America; 3 Department of Medicine, Brigham and Women’s Hospital, Boston, MA, United States of America; 4 Department of Medicine, Beth Israel Deaconess Medical Center, Boston, MA, United States of America; 5 The Desmond Tutu Health Foundation, University of Cape Town, Cape Town, Republic of South Africa; 6 Center for Global Health, Massachusetts General Hospital, Boston, MA, United States of America; 7 HIV Center for Clinical and Behavioral Studies, New York State Psychiatric Institute and Columbia University, New York, NY, United States of America; 8 Department of Medicine, Institute of Infectious Disease and Molecular Medicine, University of Cape Town, Cape Town, Republic of South Africa; 9 Governing Council, International AIDS Society, Geneva, Switzerland; 10 Institute for Collaboration on Health, Intervention and Policy, University of Connecticut, Storrs, CT, United States of America; 11 Harvard Global Health Institute, Harvard University, Cambridge, MA, United States of America; 12 Division of Women’s Health, Brigham and Women’s Hospital, Boston, MA, United States of America; York University, CANADA

## Abstract

**Aims:**

Half of young adults diagnosed with HIV in South Africa start antiretroviral therapy (ART). We developed and field tested a facilitator-guided peer support group called Yima Nkqo (“Standing Tall” in isiXhosa) to promote treatment initiation for young adults newly diagnosed with HIV in communities around Cape Town.

**Methods:**

Following an adapted version of the UK Medical Research Council’s framework for developing complex interventions, we 1) identified evidence on previous interventions to improve ART uptake in sub-Saharan Africa; 2) collected and analyzed qualitative data on the acceptability of our proposed intervention; 3) proposed a theoretical understanding of the process of behavior change; and 4) developed an intervention manual and feedback tools. During field-testing, participant feedback on intervention acceptability, and team feedback on consistency of content delivery and facilitation quality, were analyzed using an iterative, rapid-feedback evaluation approach. In-depth written and verbal summaries were shared in weekly team meetings. Team members interpreted feedback, identified areas for improvement, and proposed suggestions for intervention modifications.

**Results:**

Based on our formative research, we developed three, 90-minute sessions with content including HIV and ART education, reflection on personal resources and strengths, practice disclosing one’s status, strategies to overcome stressors, and goal setting to start treatment. A lay facilitator was trained to deliver intervention content. Two field testing groups (five and four participants, respectively) completed the intervention. Participants highlighted that strengths of Yima Nkqo included peer support, motivation, and education about HIV and ART. Team feedback to the facilitator ensured optimal consistency of intervention content delivery.

**Conclusions:**

Iteratively developed in collaboration with youth and healthcare providers, Yima Nkqo is a promising new intervention to improve treatment uptake among young adults with HIV in South Africa. The next phase will be a pilot randomized controlled trial of Yima Nkqo (ClinicalTrials.gov Identifier: NCT04568460).

## Introduction

Adolescents and young adults in South Africa have the highest annual incidence of HIV and are the fastest growing group of people in the general population living with HIV [1]. Yet only 52% of young people with HIV ages 15–24 years are on antiretroviral therapy (ART), in comparison to 71% of adults with HIV ages 25–49 and 82% of adults with HIV ages 50+ [2]. There are multiple challenges for young people diagnosed with HIV, including the need to process a new diagnosis while navigating a period of rapid physical and emotional development [3]; economic, legal, and social dependence [4]; and substantial unmet mental health needs [5]. In addition, concerns about stigma and confidentiality, clinic appointments conflicting with school and job attendance, and perceived lack of sensitivity among healthcare providers hinder treatment uptake [6].

Interventions to engage young people in HIV care in South Africa and throughout sub-Saharan Africa have targeted various points along the HIV care continuum, including prevention [7], testing [8], and retention in care [9]. Some interventions have focused on incorporating mental health care [5], community or household-based support [9], and economic empowerment [10]. Others have emphasized communication strategies [11] and adolescent-friendly clinics and treatment programs [12]. However, a recent review of HIV care interventions across the HIV care continuum for young adults in sub-Saharan Africa have found limited evidence for their effectiveness [13]. Moreover, few interventions specifically target linkage to care and treatment initiation [4, 14], even though these stages are where youth are most likely to fall off the care continuum [15]. Adolescents and young adults who aquire HIV during adolescence, as compared to those born with HIV, may especially benefit from support to promote treatment initiation in the early period after a new HIV diagnosis, at a time when successful initiation may improve long-term outcomes [4].

To respond to the critical need to support treatment initiation among young adults in South Africa, we developed the Yima Nkqo intervention. “Yima Nkqo” is a isiXhosa phrase meaning “Standing Tall.” It was inspired by the Hlanganani Program, which promoted linkage to care for young people in Cape Town through a cognitive behavioral support group [14]. Yima Nkqo consists of a three-session, facilitator-guided peer support group, designed to encourage treatment initiation for young people newly diagnosed via mobile testing sites in communities around Cape Town. In this paper, we present the four-phase process of developing this intervention. We then present lessons learned from the development process, including participant feedback on the acceptability of the field testing sessions and team feedback on the consistency of intervention delivery.

## Methods

We developed Yima Nkqo following an adapted version of the UK Medical Research Council’s framework for developing complex interventions [16]. First, we identified the best available evidence on previous interventions to improve ART uptake in sub-Saharan Africa. Second, we collected and analyzed qualitative data on the acceptability of our proposed intervention among our target population. Third, we proposed a theoretical understanding of the likely process of behavior change. Fourth, we developed the intervention manual and feedback tools. In our Results section, we describe the intervention content developed and lessons learned from team members and participants, which we used to iteratively refine the intervention for our future pilot study.

### Step 1: Formative evidence

Our extensive review of the literature and discussions with colleagues revealed only three interventions to promote ART uptake among adolescents and young adults in sub-Saharan Africa. Hlanganani HIV+ Youth Support Group sought to improve linkage to care for newly diagnosed young people ages 16–24 years old in peri-urban districts in the region around Cape Town, South Africa [14]. In three weekly sessions, Hlanganani provided cognitive behavioral therapy tools embedded into themes of coping and support, HIV health, and positive prevention. Lay facilitators with basic HIV counseling skills and experience working with youth were trained to lead the intervention in community. The pilot program showed high feasibility and acceptability, as well as improvements in disclosure and coping between baseline and follow-up after the third session. All 35 intervention participants attended their first ART visit in comparison to 58.1% of those in the comparison arm (p > 0.001).

In Kenya, a youth “Red Carpet Program” was implemented in public healthcare facilities and schools for youth newly diagnosed with HIV [4]. It provided peer counseling, psychosocial support, education, and other services to guide young adults through linkage to care and treatment. Within six months of the program, 97.3% of participants linked to care in the implementation arm (and 79% started treatment), compared to 56.5% at pre-implementation. However, the authors note that there was no contemporaneous comparison group, so they could not rule out background secular trends.

In Zimbabwe, an intervention sought to link 18–24 year-olds with HIV to treatment by using trained peer counselors [17]. This intervention was part of a nation-wide program of peer community adolescent treatment supporters (CATS), who aimed to support all youth with HIV throughout the HIV care continuum. The CATS’ broader responsibilities included linking youth to care, co-facilitating monthly support groups and ART refill groups, conducting home visits, sending SMS and phone check-ins, counseling, conducting outreach visits, and co-facilitating caregiver workshops. This particular intervention focused on identifying and referring for HIV testing the household contacts and sexual partners of young people with HIV. Of these individuals who tested positive for HIV, 96.6% were initiated on ART, and 89.9% of those were virally suppressed at six or more months. However, there was no control group in this study.

Other interventions designed to improve care engagement for adolescents and young adults with HIV have focused on adherence and viral suppression for youth already on ART rather than ART initiation. For example, ART adherence and viral suppression were higher among adolescents attending adolescent-friendly clinics in South Africa [12]. A program consisting of community-based support provided by lay health workers for adolescents receiving ART in South Africa found lower patient attrition in the intervention group versus for participants in the standard of care group [18].

The scarcity of interventions promoting ART uptake among newly diagnosed young adults highlighted the need for evidence-based interventions among this group. Of the interventions we found, we chose to adapt Hlanganani for several reasons. First, it had a control group and showed promising early findings of high linkage to care in the intervention arm. Second, it engaged youth in the greater Cape Town area, who provided a critical model for our population of youth in Cape Town. Finally, the other two interventions were broader in scope, which made them less easily translatable to our pilot intervention. The “Red Carpet Program” in Kenya was implemented across 25 schools and 50 healthcare facilities. The CATS intervention in Zimbabwe focused on supporting the overall development of youth with HIV across 24 districts in the country. Nonetheless, we found common themes across all three programs, including the emphasis on peer-led interventions and the need to be aware of potential mental health challenges among youth.

We chose to adapt rather than adopt the Hlanganani intervention in order to address some key limitations of the original intervention at the individual, social, and structural levels. At the individual level, it remained unclear–without an intention-to-treat analysis–if the intervention was truly efficacious. At the social level, the intervention only provided support for participants to initiate care, but no additional social support once a youth entered treatment. At the structural level, feasibility was limited by a remote location for sessions (only 68% completed all three sessions), and treatment was not offered in the intervention (only referral to care)–likely contributors to attrition in the care cascade. In addition, Hlanganani was piloted in 2010, before South Africa’s Universal Test and Treat guidelines were implemented in 2016, which called for first-line ART initiation among all persons with HIV regardless of CD4 count [19]. We therefore chose to adapt Hlanganani to inform the socio-behavioral components of our proposed treatment initiation intervention among young adults newly diagnosed around Cape Town.

### Step 2: Qualitative study

To elicit perceived barriers to treatment initiation and the acceptability of our proposed Yima Nkqo intervention, our team conducted qualitative semi-structured interviews with youth ages 18–24 from July 2018 to May 2019. These youth were ART-naïve, meaning that they reported not previously accessing ART. Details of the study design have been published [20]. Briefly, we recruited and enrolled a convenience sample of 20 young adults and 10 healthcare workers in four communities around Cape Town (Philippi, Crossroads, Mitchells Plain, and Dunoon). Sites of recruitment included two mobile “Tutu Tester” vans, which circulate throughout these four communities to provide HIV testing to youth, and community projects offering youth HIV testing and health screening in the same areas. Interviews were conducted with participants using a qualitative semi-structured guide, with one guide for young adults and one guide for healthcare workers. The guides sought to elicit the anticipated barriers to starting treatment for newly-diagnosed youth. For example, the interview guide for youth asked, “What might be some reasons people diagnosed with HIV would not start treatment?” Interviewers also sought ideas for and feedback on potential intervention strategies. Questions in both guides included: “If you could design a program to help young people newly diagnosed with HIV to start treatment, what would that look like?” and “What do you think of getting medications outside of the usual clinical setting?”

Our codebook was initially organized into two sections, treatment barriers and feedback on potential intervention strategies. We iteratively developed our codebook using an inductive analytic approach informed by grounded theory [21]. Interviews were coded in Dedoose by two teams, with interrater reliability testing resulting in a pooled Cohen’s kappa of 0.86. We maintained an audit trail of recordings, transcripts, notes, and coding.

Team-based coding and analysis of the qualitative data identified three primary categories of barriers to ART initiation among young people. The first was stigmatizing social norms. Participants described the social expectation of young people being healthy. In this context, a diagnosis of HIV feels discordant with those social norms. Thus, there is a fear of potential unwanted disclosure to peers, family members, and partners, who could be unsupportive. The secondary primary category also related to anticipated stigma, but focused especially on contextual issues in the clinic rather than anticipated stigma from staff. Young people felt that the clinic’s physical layout and procedures could lead to unwanted disclosure. For example, participants reported separate waiting areas and differently colored medical folders for persons living with HIV. The last primary category focused on challenges coping, including struggles with denial, alcohol, anxiety, depression, and suicidal ideation, making it extremely difficult for youth to accept their diagnosis, let alone start treatment. Lastly, participants identified three features of the proposed Yima Nkqo intervention as most important: 1) peer support to remind youth they are not alone; 2) facilitator support to help youth develop positive coping skills and to provide HIV education; and 3) a community space that is welcoming to youth with food and music. Participants also highlighted the importance of emphasizing confidentiality within the group to avoid unwanted public disclosure.

### Step 3: Theory of change

**Fig 1:**
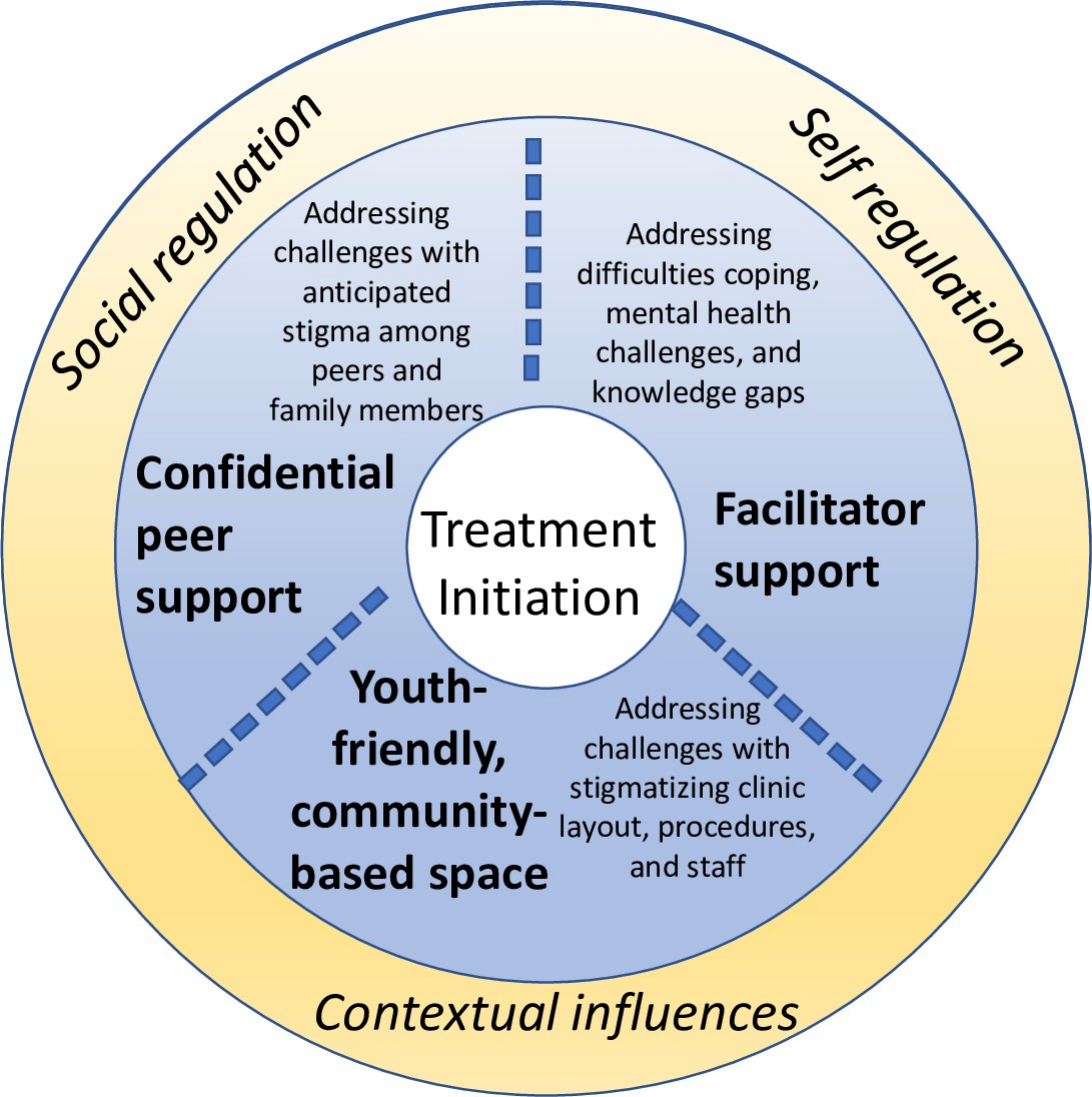
Theory of change for Yima Nkqo. Theoretical framework modified from Social Action Theory to predict how the key components of Yima Nkqo may facilitate behavior change to lead to treatment initiation for young people newly diagnosed with HIV.

Both our qualitative analyses and the development of Yima Nkqo were guided by Social Action Theory (SAT), which emphasizes that self-regulation, social regulation, and contextual factors guide health behavior [22]. It has been used in several HIV-related studies for youth and adults with HIV, including in South Africa [23]. Our qualitative findings led to the development of an explanatory framework, adapted from SAT, to explain the likely process of care engagement and treatment initiation for young people. As described previously [20], we found that barriers to treatment initiation fell within three SAT domains: stigmatizing social norms (social regulation), anticipated stigma related to clinic layout and procedures (contextual influences), and challenges coping (self-regulation). At the same time, as shown in Fig 1, our explanatory framework shows how Yima Nkqo may address these barriers and facilitate behavior change through each SAT domain, including peer support and participant confidentiality (social regulation), a youth-friendly community space (contextual influences), and facilitator support to improve coping skills (social regulation and self-regulation) [Fig 1].

### Step 4: Yima Nkqo manual development

Following our qualitative study and the development of our theory of change, the development of Yima Nkqo began in June 2019. Team members, including the study coordinator (ER), research assistants and qualitative interviewers (RJ, SS), intervention facilitators (NS, PF), and qualitative analysis leader (MFN) convened in Cape Town for two full days of participatory workshops, with additional support and guidance from intervention design leader (LB) and study PIs (ITK, LGB). The team’s goal was to synthesize the formative work to date in order to co-design a draft of the Yima Nkqo intervention manual, focusing on both intervention skills and intervention content. To guide this work, we drew on facilitator communication skills described in prior group health and wellbeing interventions [24]. We also found examples of potential group activities used in interventions to promote antiretroviral initiation for adults [25]. Example activities and topics, such as role plays on disclosure of HIV status, a mindfulness exercise to manage stress, and personal reflection on behavior change goals, were shared among team members and generated ideas for additional topics and activities. Team members also discussed and practiced facilitator communication skills to align on best practices and brainstorm strategies to support the Yima Nkqo facilitator, a lay counselor who would be trained to learn the intervention material.

### Ethics approval

This study was approved by the Human Subjects Committee at Partners Healthcare (Protocol number 2018P000961) and the University of Cape Town Human Research Ethics Committee (219/2018).

## Results

### Intervention content

By the end of the participatory workshops, a broad outline of each weekly 90-minute session of Yima Nkqo was developed, including a proposed theme and associated activities for each session. Session One focused on welcoming participants to the group and reviewing HIV basic knowledge, with activities including team-building exercises, opportunities for participants to ask questions and dispel HIV treatment myths, and creative drawing for participants to reflect on their personal resources and experiences with HIV. Session Two focused on participants’ identities and support systems, exploring the ways in which coping strategies (e.g., relaxation exercises) and disclosure to peers and family members (e.g., role-playing these discussions) could help ease the transition to ART. Session Three focused on empowering participants to start treatment through overcoming stressors, goal setting, and role playing what it would be like to go to clinics for treatment. Over the course of the next six months, further details of the intervention manual emerged through feedback from the facilitators and full team. Each of the three sessions included time for a welcome and check-in; elicitation of participants’ feelings, questions, and experiences; facilitator-guided new learning; “action” activities to practice new learnings; and reflection. Activities were designed to be highly interactive. The facilitator relied primarily on oral rather than textual communication to be accessible to those with limited literacy. More detail on the content of each session are shown in Table 1.

**Table 1:** Yima Nkqo intervention manual. Topics covered within each of the three 90-minute sessions.

	Session 1	Session 2	Session 3
**Welcome and Check-In**	• Group introductions • Development of ground rules • General overview of Yima Nkqo	Re-introductions and welcome to new members	Welcome and congratulate participants for making it to the last session as a group
**Elicitation**	“Burning Questions” about HIV and ART	“Public service announcement” about HIV treatment to elicit questions or concerns from the prior week	Group discussion to elicit stressors related to living with HIV and strategies to cope
**New Learning**	“Tree of Information” to explore facts and myths about HIV and ART	Skits about anticipating barriers to starting ART and ways to overcome them	Small group activity to compile lists of coping strategies
**Action Activities**	Group “body mapping” drawing activity to identify personal and group resources	• Continue group “body mapping” to identify personal strengths and possible support partners • Role plays to practice HIV status disclosure	Goal setting activity to clarify and discuss HIV treatment goals with a partner
**Reflection**	Deep breathing with reflection on the day’s learnings	Deep breathing with reflection on the day’s learnings	Deep breathing with reflection on the day’s learnings

Prior to the start of field testing, the facilitators (one main facilitator, one backup facilitator) familiarized themselves with Yima Nkqo materials and practiced delivering the sessions. Drop-in sessions with the Yima Nkqo facilitator were planned for participants to be able to check in and discuss any topics on their mind after the conclusion of the sessions.

### Feedback tool development and analysis

We created a feedback form for participants to complete after each field testing session, with the option of writing responses or providing verbal feedback. Open-ended questions included the following: 1) What did you like about today’s session? 2) What did not work for you today? 3) What are important things you think we did not cover? 4) Did you like where the group met? How about the time of day and days of the week? 5) Based on these 3 sessions, what will help keep young people coming every month? 6) What other supports should be in place to help young people start and stay on ARVs?

In addition, we created a form for team members to observe the faciliator to ensure consistency in intervention content delivery. The first part of this form included ten questions about the content elements of the sessions, e.g., whether the facilitator introduced the agenda, each objective, and the purpose of Yima Nkqo; elicited questions, evaluated current knowledge, and gave information about HIV/ART; and led each activity for the session. These questions had an option to respond “Yes,” “No,” “Partially,” and “N/A.” The second part of this form included 14 questions about the quality of the facilitation, including how well the facilitator appeared familiar with materials, engaged with those struggling to interact, used reflected listening, encouraged interaction between participants, and used open-ended questions. Responses to these questions were on a scale from 1 (poor/never) to 7 (excellent/always). Scores were rated as “High” when all content element questions received a “Yes” and all quality of facilitation questions received a score of 6 or 7. Scores were rated as “Adequate” when all content element questions received a “yes” and all quality of facilitation questions received a score of 4 or 5.

Feedback was analyzed using an iterative, rapid-feedback evaluation approach [26–28]. Directly following each field testing session, written feedback forms were shared with all team members on a secure platform, and in-depth, verbal summaries were shared on weekly, hour-long team meetings. In these meetings, team members discussed and analyzed feedback, identified areas for improvement, and proposed suggestions for intervention changes or targeted facilitator training. These suggestions were incorporated into the following week’s field testing session. Upon completion of the full, three-session field test for each group, a summary report of strengths, areas of growth, and suggestions for each session was compiled and shared with all team members.

### Field testing Yima Nkqo

In January and February 2020, two groups participated in Yima Nkqo field testing sessions. Participants were recruited for field testing at the same HIV testing vans where they were recruited for the qualitative interviews. The first group had five participants and the second group had four participants. All participants were ART-naïve. Each participant attended all three 90-minute weekly sessions. As described above, we obtained open-ended, written or oral feedback from participants after attending each session as well as feedback by a Yima Nkqo research assistant (SS) who observed the sessions to ensure consistency in intervention content delivery.

#### Participant feedback

Participants highlighted three strengths of Yima Nkqo: sharing with and learning from peers with HIV; the sense of motivation and support from the facilitator; and education about HIV and ART. One participant wrote, “I will stop having bad thoughts about myself because I now have people that support me.” Another participant raised a mental health concern, saying, “I am always having dark thoughts,” but “I love everything that we are doing here, and I wish I was always here.” The facilitator received additional training in recognizing the need for referral to mental health services, and all participants received a packet with information about local mental health resources. In response to participant feedback, we increased the amount of nutritious refreshments that were provided to participants during each session in recognition that many participants experienced food insecurity.

#### Team feedback on consistency of intervention delivery and facilitator training

The facilitator was consistent in delivering intervention content material, scoring “yes” in areas such as encouraging participants to ask questions, assessing participants’ current HIV knowledge, clearly presenting information regarding HIV and ART, and summarizing the sessions. The faciliator received maximum scores in skillfully helping participants to think through their decision-making skills, using reflective listening, encouraging question-asking and interaction among participants, and using open-ended questions. Additional support was provided to the facilitator to enhance her comfort with the material through training sessions with a local intervention specialist as well as practice sessions with colleagues. In addition, some activities were slightly modified in order to make the intervention easier to deliver within the 90-minute session. For example, the body mapping exercise was changed such that rather than each participant doing their own individual drawing to share with the group, the participants did a shared “body map” drawing together.

## Discussion

We used an evidence-based, iterative process to develop a novel intervention designed to support treatment uptake among adolescents and young adults newly diagnosed with HIV. Participant feedback in field testing was positive and emphasized the importance of peer support from other youth with HIV as well as the motivation they derived from these peers and the facilitator. This feedback confirmed similar findings from our formative qualitative work as well as our model of behavior change. It also builds on a small but growing number of HIV program acceptability studies with adolescents and young adults in sub-Saharan Africa [29]. Similar to this research, we found that offering social support within a safe, stigma-free environment is highly acceptable to youth with HIV [14, 30]. Such an environment allows youth to feel comfortable discussing personal challenges and developing trusting relationships, thus motivating them to accept their HIV status [29, 31]. While our field testing did not evaluate ART uptake, there has been evidence showing the efficacy of peer support on ART outcomes. For example, a study examining a peer-based intervention among South African pregnant women with HIV showed positive effects for ART adherence and retention [32].

In addition to peer support and motivation within a stigma-free environment, field testing participants appreciated the educational content related to HIV and ART. Research to promote ART initiation among adolescents and young adults has largely focused on the psychosocial and structural challenges known to create barrires to care, including mental health challenges and a perceived lack of adolescent-friendly and confidential services in health facilities [33]. At the same time, research to examine HIV literacy among adolescents who acquired HIV at birth has revealed meaningful gaps in treatment literacy that impact disclosure and HIV care [33, 34]. Similar research among adolescents who acquired HIV later in life is scarce. Related studies on the development of mobile health interventions to prevent HIV among young adults in sub-Saharan Africa have similarly found acceptability of the knowledge-sharing components of these interventions [35, 36].

Participant feedback highlighted that mental health concerns remain challenges for young people. Research has shown that adolescents living with HIV are at heightened risk for mental health challenges as compared to their peers, which may negatively impact HIV care outcomes [37]. There is also evidence that resilience, depression, and self-efficacy are significant mediators between HIV-related stigma and health outcomes [38], and in particular, higher depression and anxiety symptoms have been associated with higher internalized stigma [39]. Therefore, addressing youth’s mental wellbeing may also mitigate the impact of HIV-related stigma. In sub-Saharan Africa, mental health interventions for people with HIV have included psychological and pharmacological treatments, family strengthening, and economic empowerment interventions, but these efforts are often limited by a lack of funding and available professionally trained staff, and there is a paucity of such interventions among youth [40]. There is also a gap in studies which assess the acceptability of mental health interventions for adolescents [29]. Recognizing this key issue through our formative work, our intervention was designed to include activities designed to support mental health, e.g., social connection and personal reflection through deep breathing. We also identified and promptly referred participants requiring professional mental health services beyond the scope of our intervention.

Some participants raised the importance of refreshments and reimbursements for travel in our intervention. Food insecurity remains a major issue in some communities in South Africa and has a negative impact on HIV treatment engagement, such as declining free ART in South Africa for fear of taking ART on an empty stomach [41]. Studies have shown that providing nutritional support can minimize the harmful effects of food insecurity on HIV outcomes [42, 43], and we also found that it was a factor in participants’ willingness to attend the intervention sessions. Therefore, we made sure to include sufficient refreshments in the sessions. Future scale up of this or similar interventions may also benefit from providing some level of nutritional support. Budgeting for this upfront may help avoid later costs of tracking individuals who are lost to follow up or require more costly medical care. More broadly, poverty impedes HIV care engagement [44] and is a key social determinant of mental health [45]. However, evidence is mixed on the best way to address poverty to promote ART uptake. Some research in sub-Saharan Africa has shown that economic incentives are effective in promoting ART adherence [46], whereas an economic incentive intervention for adults in Cape Town diagnosed via a mobile health clinic did not show an impact in ART uptake [47].

Training programs for facilitators involved in HIV interventions have emphasized building skills in peer support and self-care, as well as becoming comfortable understanding and delivering essential knowledge [48]. Therefore, ongoing support for the faciliator to familiarize herself with intervention content and skills was critical to our intervention development process. We found that useful strategies to support the faciliator included expert training and peer observation of practice sessions by colleagues. At the same time, research on participatory HIV interventions in South Africa has identified that facilitators’ success is influenced not only by these factors but also by important broader contextual factors [49]. These factors include the extent to which faciliators have been shaped by their own educational background in didactic versus participatory settings, and whether facilitators are motivated by “softer” measures of critical thinking and reflection among participants, which often underpin the theoretical drivers of behavioral change [50]. Therefore, we focused on training the facilitator to foster participant engagement and reflection.

SAT guided both our qualitative analyses as well as our intervention development process by shaping our understanding of the multi-level factors which influence young people’s engagement in care. We originally hypothesized that Yima Nkqo would address address barriers to HIV care and facilitate behavior change through each SAT domain, including peer support and participant confidentiality (social regulation), a youth-friendly community space (contextual influences), and facilitator support to improve coping skills (self-regulation). Our data show that each intervention component did not solely address a single domain, as hypothesized, but rather there was inherent overlap and interplay between domains. For example, an important role of the facilitator was to help participants to develop positive coping skills and to share knowledge with them, which contributed to participants’ self-regulation processes. However, facilitators also played a role in setting a positive and supportive tone to create a space that felt stigma-free, which enabled participants to share confidential challenges comfortably (social regulation).

We were limited by scheduling difficulties given participants’ varied school, work, and family schedules, which could create barriers to participation. However, our facilitator contacted every participant before each session to ensure attendance, and all participants in our field testing came for each session. Additionally, themes discussed during the intervention sessions may elicit strong emotions, which can be challenging for the facilitator and the participants. We trained the facilitator to recognize and support participants at these times as well as recognize when referral to a mental health professional would be necessary. Finally, given that this intervention was iteratively developed for a specific population and context, it may not be generalizable. At the same time, we did observe consistency in parts of our intervention that resonated with participants and with what has been shown to be effective in HIV interventions in other contexts, e.g., peer support.

## Conclusions

Strategies to support young people in starting ART are urgently needed in South Africa. Yima Nkqo is a new intervention to support ART initiation that has been iteratively developed in collaboration with young adults and the healthcare providers who serve them. Early lessons from our field testing suggest that the intervention content can be delivered consistently by a trained lay faciliator and that participants appreciate the support, motivation, and education they received. The next phase of research will be a pilot randomized controlled trial of Yima Nkqo to assess acceptability and feasibility of this intervention.

## Supporting information

S1 File Inclusivity in global research. (DOCX)
